# Bibliometrics and knowledge map analysis of ultrasound-guided regional anesthesia

**DOI:** 10.1515/med-2023-0813

**Published:** 2023-10-13

**Authors:** Gao Manhai, Wei Wei, Hao Xiaolu, Wu Juan

**Affiliations:** Department of Anesthesia Surgery, The First Affiliated Hospital of Baotou Medical College, Inner Mongolia University of Science and Technology, Baotou, 014010 China

**Keywords:** bibliometrics, ultrasound-guided, regional anesthesia

## Abstract

Through bibliometric analysis, we aim to comprehensively understand the research dynamics in this field, reveal key scientific research achievements and breakthrough discoveries, and provide valuable reference and guidance for future research directions. Utilizing the Web of Science, we retrieved the literature pertaining to ultrasonics-guided regional anesthesiology (1994–2022). CiteSpace and VOSviewer were used for bibliometric and knowledge mapping analysis. Our examination encompassed publication trends, authorship patterns, institutional contributions, frequently occurring keywords, keyword clustering, and emerging terminology trends. Of the 570 papers reviewed, there was a rising trend in publications each year. The main keywords in regional anesthesia were ultrasound guidance, nerve, analgesia, and pain score. Key research areas were regional anesthesia, ultrasound guidance, approach, pain score, and plane block. The U.S. led in research. Stanford University, University of Toronto, and Cork University Hospital were central institutions. Chan V was the top author with 24 articles, while Marhofer P was the most cited at 150 times. Regional anesthesia and pain medicine were the predominant journal in both publications and citations. In conclusion, research in this field consistently grew yearly, and visualization showcased trends in ultrasound-guided regional anesthesia. These visuals provided key bibliometric insights, helping researchers further explore and understand this domain.

## 1 Introduction

Efficient anesthesia assistance plays a pivotal role in alleviating postoperative pain and facilitating a swift recovery process [1]. The fundamental principle in the selection of an anesthesia method is to not only attain the necessary depth of anesthesia for the surgical procedure but also prioritize anesthesia safety. Numerous studies have highlighted the importance of allowing patients a period of awakening after general anesthesia. During this phase, postoperative functional recovery tends to be sluggish, potentially giving rise to a range of postoperative complications [2]. In this context, regional nerve analgesia plays a crucial role in mitigating respiratory and circulatory disturbances, effectively broadening the scope of anesthesia indications while concurrently lowering the likelihood of postoperative complications [3].

The key to regional nerve block analgesia is to give sufficient and safe local anesthesia to the target area. Ultrasound-guided regional anesthesia employs real-time ultrasound imaging to visualize soft tissue anatomical structures, thereby facilitating the precise and rapid localization of the target anesthesia site, enhancing both safety and efficacy. This enables the surgeon to precisely block the specific nerves responsible for motor and sensory functions based on the lesion’s location, thereby fulfilling the surgical requirements [4]. It significantly enhances the precision and success rate of anesthesia while also reducing the overall anesthesia usage when compared to empirically guided nerve blocks or nerve stimulator-guided nerve blocks [5–7]. Researchers are constantly exploring new areas and applications, such as spinal and plexus block anesthesia, and improving postoperative pain management. More and more research focusing on individual differences to develop more personalized anesthesia plans and drug choices, improve anesthesia accuracy and patient safety, reduce complications, and provide more predictable outcomes has important implications for the medical field. Although ultrasound-guided regional anesthesia is a safe and effective technique, a number of contraindications remain, including but not limited to, infection or skin damage, bleeding, allergic reactions, nerve damage, etc. Ultrasound-guided regional anesthesia is widely used in surgery and pain management and has developed rapidly in recent years.

Given its advantages in terms of convenience, visibility, and safety, there is a substantial body of literature dedicated to ultrasound-guided regional anesthesia. To effectively harness and analyze this pertinent data and consequently investigate the developmental landscape of this field, identify key research focal points, and address existing limitations, this study employed CiteSpace and VOSviewer software for bibliometric analysis of relevant literature in the realm of ultrasonics-guided regional anesthesia from 1994 onwards, as indexed on Web of Science. Through bibliometric analysis, we aim to provide in-depth insight into scientific research and clinical practice in the field, drive the further development of knowledge, and promote progress and innovation in the medical field. Bibliometric analysis, as a popular and rigorous method, will help reveal the academic landscape of the field and provide a more solid foundation for related research.

## 2 Materials and methods

### 2.1 Data sources

In this study, Web of Science (https://www.webofscience.com/) was selected as the literature retrieval platform and the core collection database of Web of Science was selected. Used as “Ultrasound – Guide (Title) AND regional anesthesia (Topic) AND Article Document Type NOT corpse (Abstract) “Cadaver* (Abstract) NOT dog (Abstract) NOT cat (Abstract) NOT feli* (Abstract)” was selected as the retrieval term, animal experiments and cadaver-based studies were excluded, and Article was selected as the literature type. A total of 570 results were obtained by searching articles published by relevant scholars from 1900 to 2022.

### 2.2 Research methods

To realize in-depth research and analysis of ultrasound-guided regional anesthesia, two researchers were selected to download the data, and the basic information of the included literature was exported and saved (including the author, publication institution, publication quantity, keywords, title of article, name of source journal, abstract, and other information). Visual analysis software was used to analyze the information of research topics, hot spots, authors, institutions, keywords co-occurrence and cluster analysis, emergence words, and other information.

### 2.3 Analysis tools

All valid data were collected in Web of Science core collection and imported into Microsoft Excel 2016, VOSviewer1.6.10, and CiteSpace6.1.R2 for visual analysis.

CiteSpace is a visualization software for bibliometric analysis using the Java platform [8]. It is an interactive analysis tool that realizes the visualization task of scientific mapping by combining bibliometrics, visual analysis methods, and data mining algorithms [9]. CiteSpace provides a variety of functional options for bibliometrics, including collaborative network analysis, co-citation analysis, and co-occurrence analysis, and can generate visual maps. Nodes with high centrality (>0.1) in the visualization map are often considered turning points or key points in a domain. When the default number of CiteSpace nodes is greater than 350, centrality computing is disabled. We need to manually click on the “Compute Node Centrality” feature in the node menu.

VOSviewer is an application for building and viewing bibliometric maps. It can be used to build author or journal maps based on collaborative data, or keyword maps based on co-occurrence data. The project provides a viewer that allows for a comprehensive and detailed review of bibliometric maps. Unlike common bibliometrics programs, VOSviewer focuses specifically on graphical representation of bibliometrics, which is particularly useful for displaying large bibliometrics in an easily interpreted manner. VOSviewer can analyze bibliometrics networks, build visual network diagrams, and ultimately achieve a deep and comprehensive understanding of the structure and dynamic development of scientific research. The font size of VOSviewer map node represents the frequency of its occurrence. Each point in the project density visualization has a color that represents the project density for that point. The greater the number of items near a point, the higher the weight of adjacent items, the closer the color of the point to yellow, and *vice versa*, the closer the color of the point to blue. Different colors of nodes indicate that they belong to different clusters [10].

Microsoft Office Excel 2016 was used to analyze the trend of the number of articles published in the year.

## 3 Results

### 3.1 Chronological distribution of literature publication

**Figure 1:**
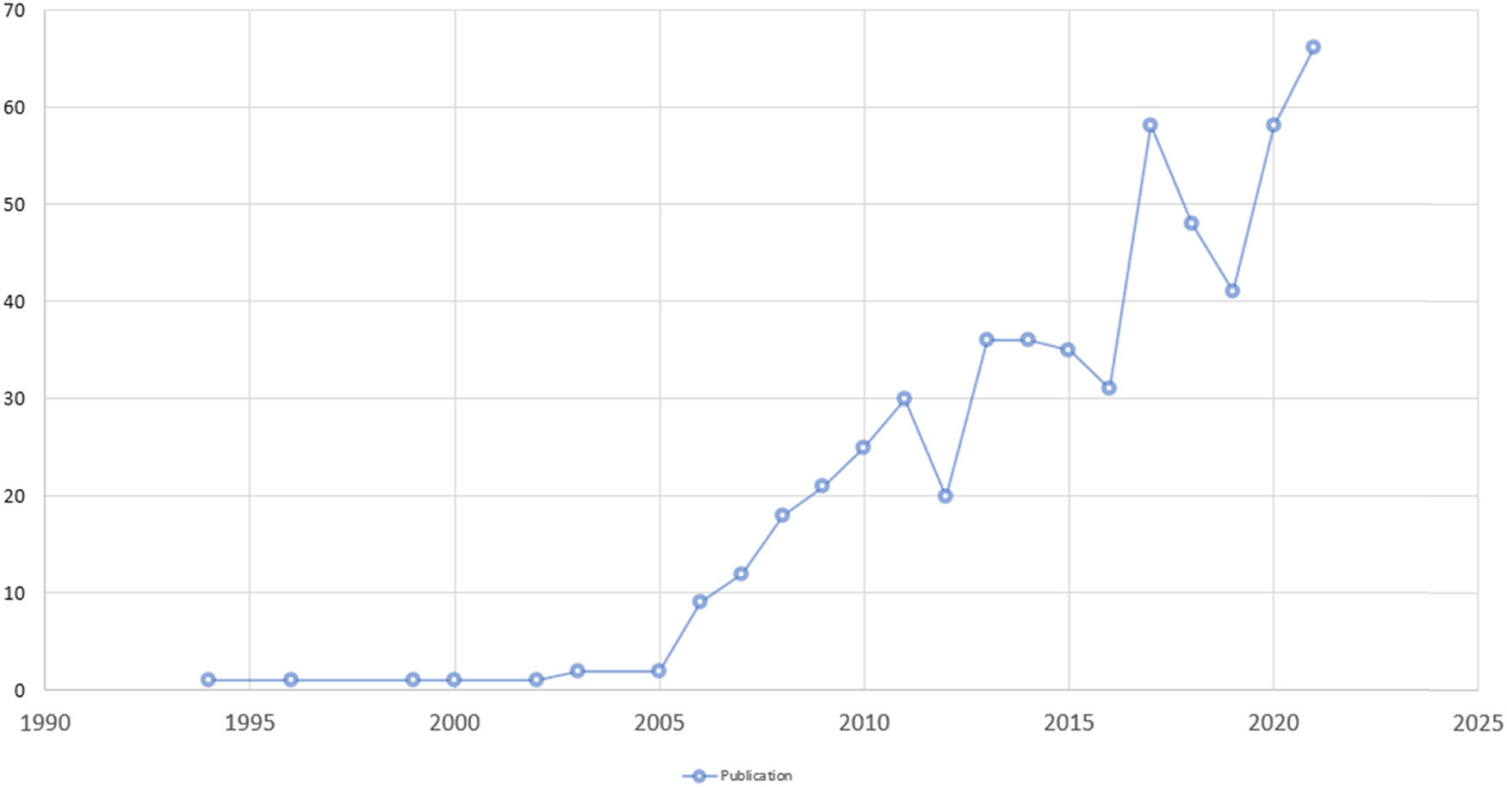
Volume and year of ultrasound-guided regional anesthesia.

Statistical analysis was conducted on the temporal distribution of all the included literatures, and the publication temporal distribution of literatures related to ultrasound-guided regional anesthesia research was obtained, as shown in Figure 1.

According to the statistics on the number of articles published in the included literature, the literature on ultrasound-guided regional anesthesia first appeared in 1994, but there was only one literature in that year. Before 2006, the annual number of articles was basically stable, and after 2007, it showed a rapid growth. In general, in recent years, the related researches in the field of regional anesthesia under the guidance of ultrasound have been increasing year by year, and the research in this field has been gradually concerned by more and more scholars.

### 3.2 Times cited and publications over time

**Figure 2:**
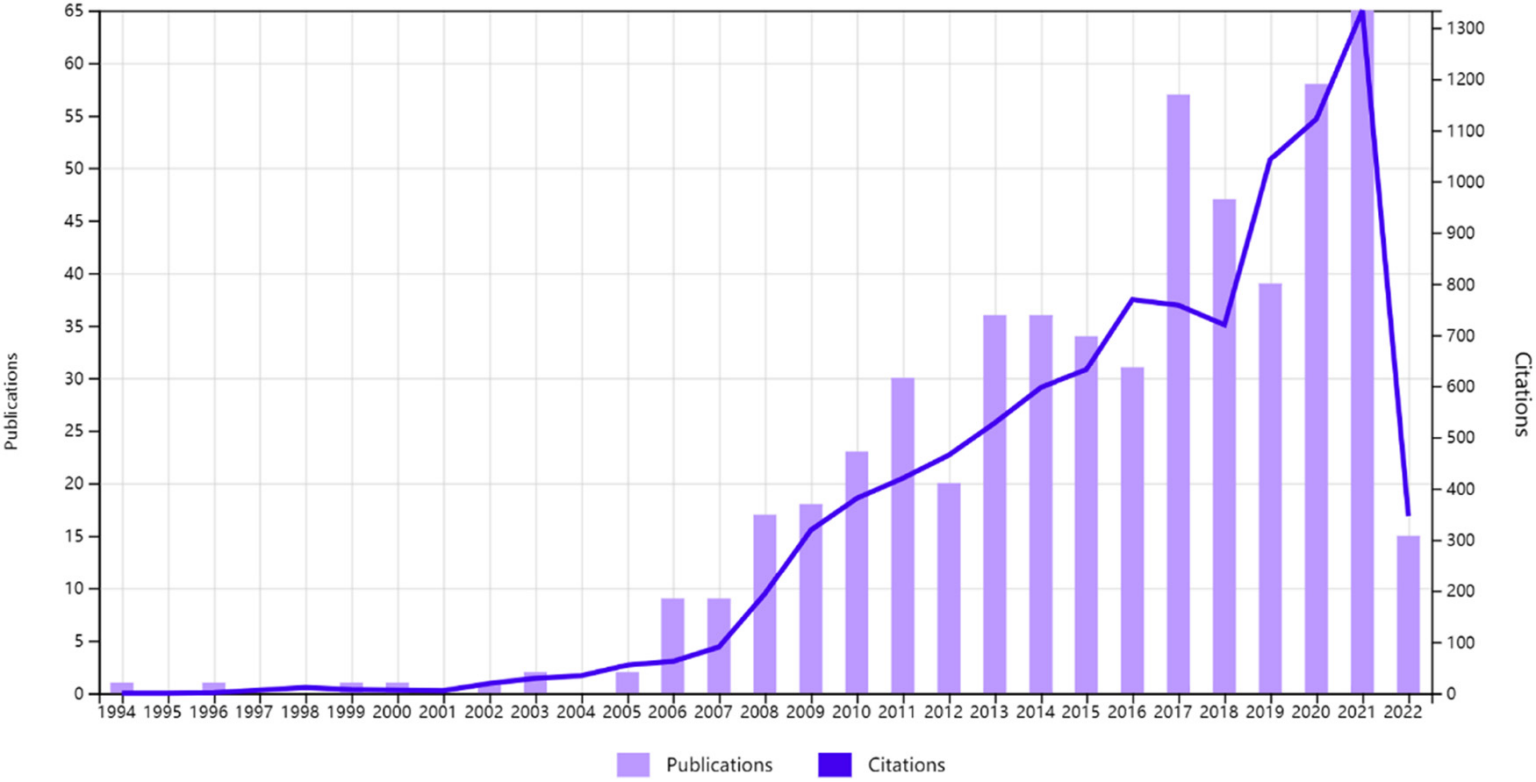
Times cited and publications over time.

The 570 references retrieved according to the subject words were cited 9,953 times in total, and 8,901 times excluding self-citation, with an average of 18 times per paper. As can be seen from Figure 2, the number of articles citing literatures in this field has gradually increased since 2006. Citation frequency statistics were performed on the retrieval results. The top 10 references in citation frequency in the field of ultrasound-guided regional anesthesia are listed in Table 1. Literatures with high citation frequency were mainly published after 2007. The title, published in 2018, is “Ultrasound-Guided Transversus Abdominis Plane Block: description of a new technique and comparison with conventional systemic analgesia during laparoscopic Reference “cholecystectomy” was cited the most frequently (258 times), with an average of 18.43 times per year. This article mainly discusses the effect and prospect of ultrasound-guided regional anesthesia.

**Table 1:** Top 10 cited references

	Title	Year	Total citations	Average per year
1	Ultrasound-guided transversus abdominis plane block: description of a new technique and comparison with conventional systemic analgesia during laparoscopic cholecystectomy	2009	258	18.43
2	Ultrasound-guided supraclavicular approach for regional anesthesia of the brachial-plexus	1994	234	8.07
3	Characterizing novice behavior associated with learning ultrasound-guided peripheral regional anesthesia	2007	222	13.88
4	Ultrasound-guided infraclavicular brachial plexus block	2002	207	9.86
5	Ultrasound-guided supraclavicular brachial plexus block	2003	181	9.05
6	Needle visualization in ultrasound-guided regional anesthesia: challenges and solutions	2008	178	11.87
7	Ultrasound-guided regional anesthesia: Current concepts and future trends	2007	173	10.81
8	Incidence of local anesthetic systemic toxicity and postoperative neurologic symptoms associated with 12,668 ultrasound-guided nerve blocks	2012	163	14.82
9	Ultrasound guided erector spinae plane block reduces postoperative opioid consumption following breast surgery: a randomized controlled study	2018	145	29
10	The ASRA evidence-based medicine assessment of ultrasound-guided regional anesthesia and pain medicine executive summary	2010	134	10.31

### 3.3 Keywords co-occurrence analysis

**Figure 3:**
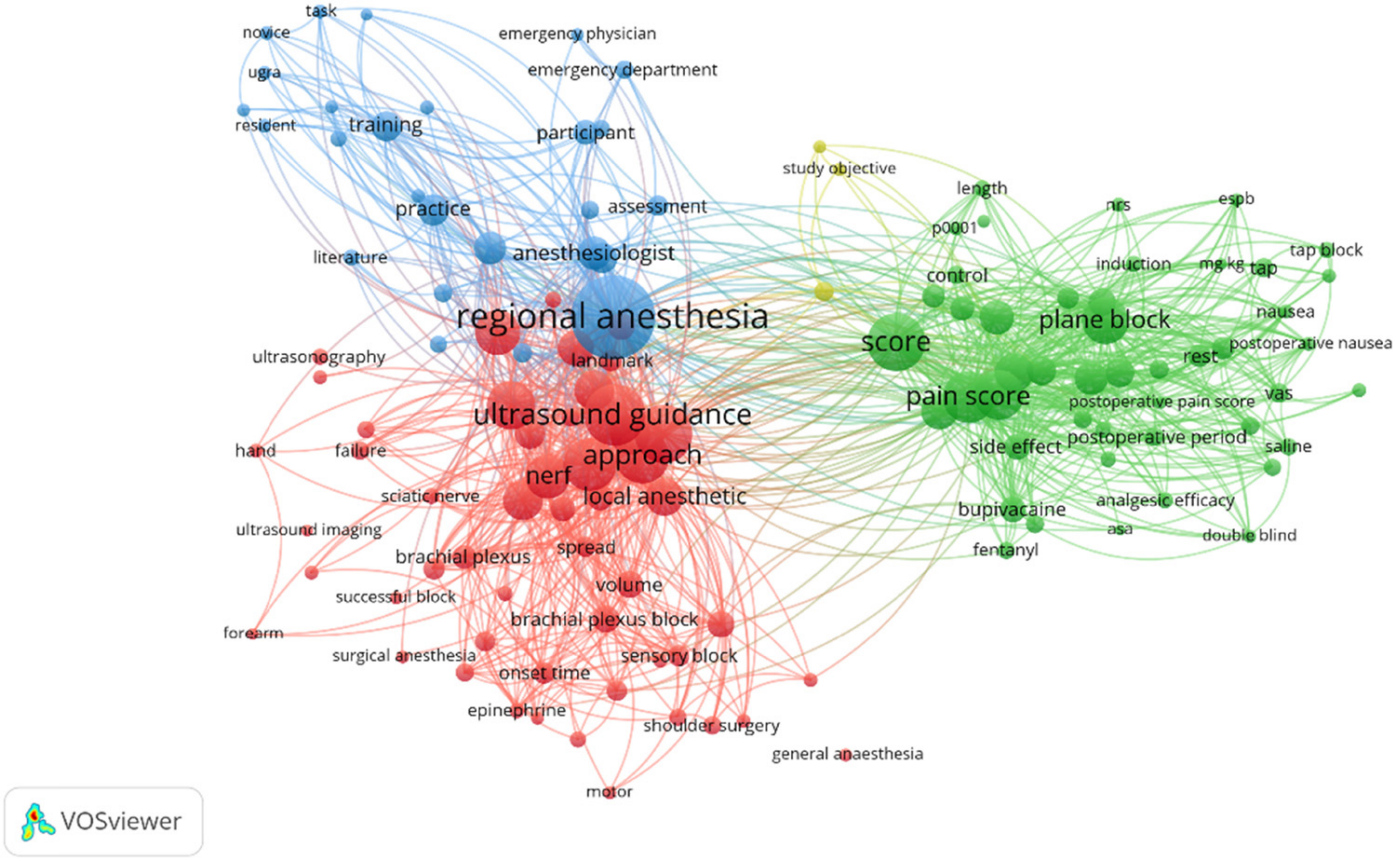
Keywords co-occurrence analysis.

**Figure 4:**
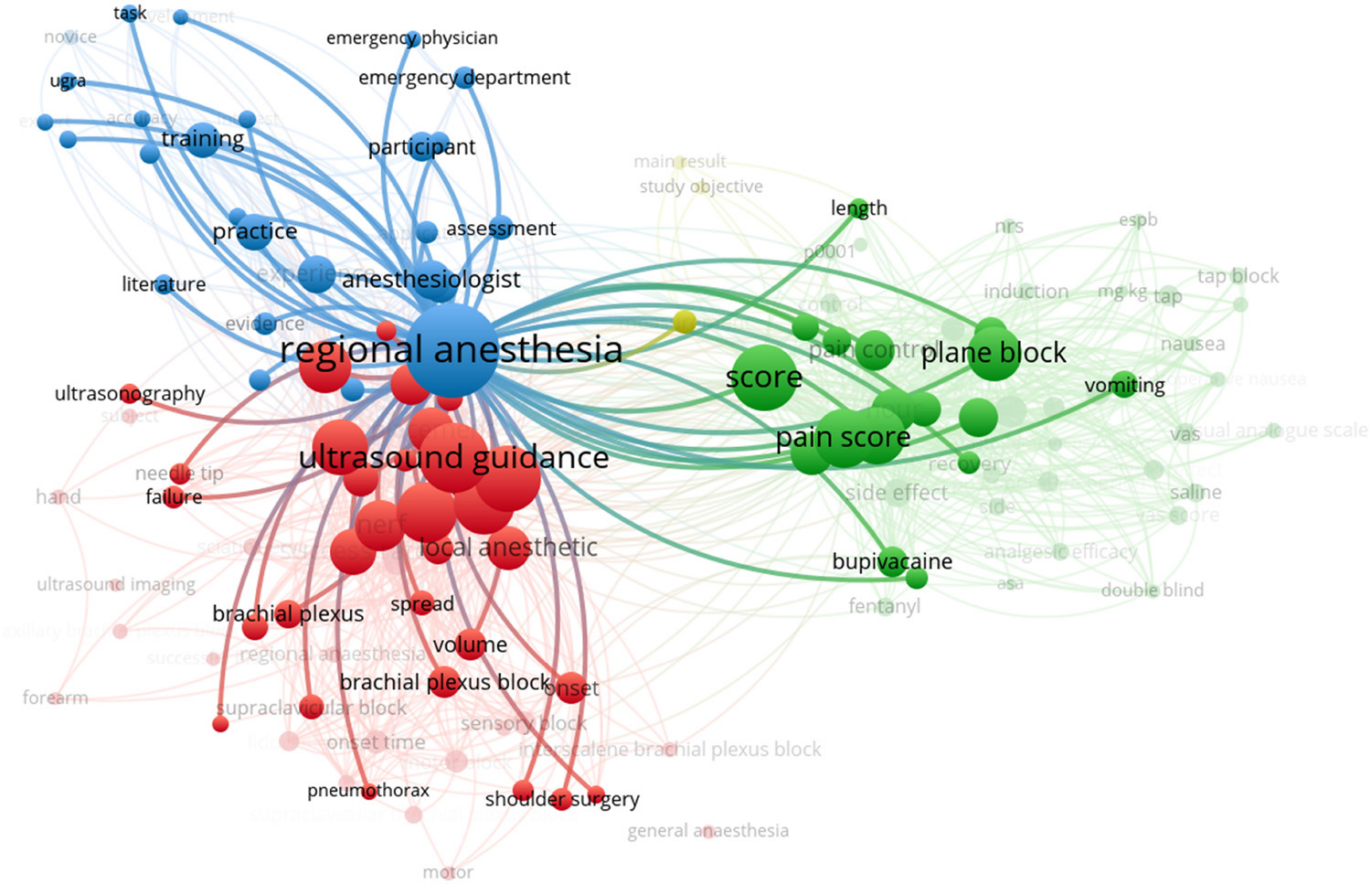
Keywords with the most occurrences.

VOSviewer software was utilized to generate a visualization map depicting research in the field of regional anesthesia guided by ultrasound. In this map, the font size of keywords corresponds to the frequency of their occurrence, and the primary emphasis is on keyword clustering. Figure 3 exhibits four distinct clusters, encompassing a total of 128 keywords and 5,748 connections. These clusters are color-coded, with the blue cluster featuring 26 keywords, the green cluster comprising 47 keywords, the yellow cluster containing 3 keywords, and the red cluster including 52 keywords. Figure 4 highlights that regional anesthesia emerges as the most frequently occurring keyword, with 126 related keywords associated with it. Notably, the key terms closely associated with regional anesthesia include ultrasound guidance, nerves, analgesia, and pain scores. Table 2 shows the top 10 keywords in terms of frequency and mediating centrality. Regional anesthesia, guidance, and pain are the top 3 in both orders, indicating the importance of these three words.

**Table 2:** Top 10 keywords in terms of number of publications

	Count	Year	Keyword		Centrality	Year	Keyword
1	270	1999	Regional anesthesia	1	0.35	1999	Regional anesthesia
2	116	2003	Guidance	2	0.3	2003	Guidance
3	78	2006	Pain	3	0.2	2006	Pain
4	73	2009	Analgesia	4	0.17	2006	Brachial plexus block
5	70	1999	Surgery	5	0.14	2009	Analgesia
6	62	2006	Brachial plexus block	5	0.14	2002	Anesthesia
7	50	2002	Anesthesia	7	0.13	1999	Surgery
8	45	2006	Children	8	0.11	2008	Complication
9	42	2008	Complication	8	0.11	2009	Nerve block
10	39	2009	Nerve block	10	0.07	2006	Children

### 3.4 Research hot spot tracking

**Figure 5:**
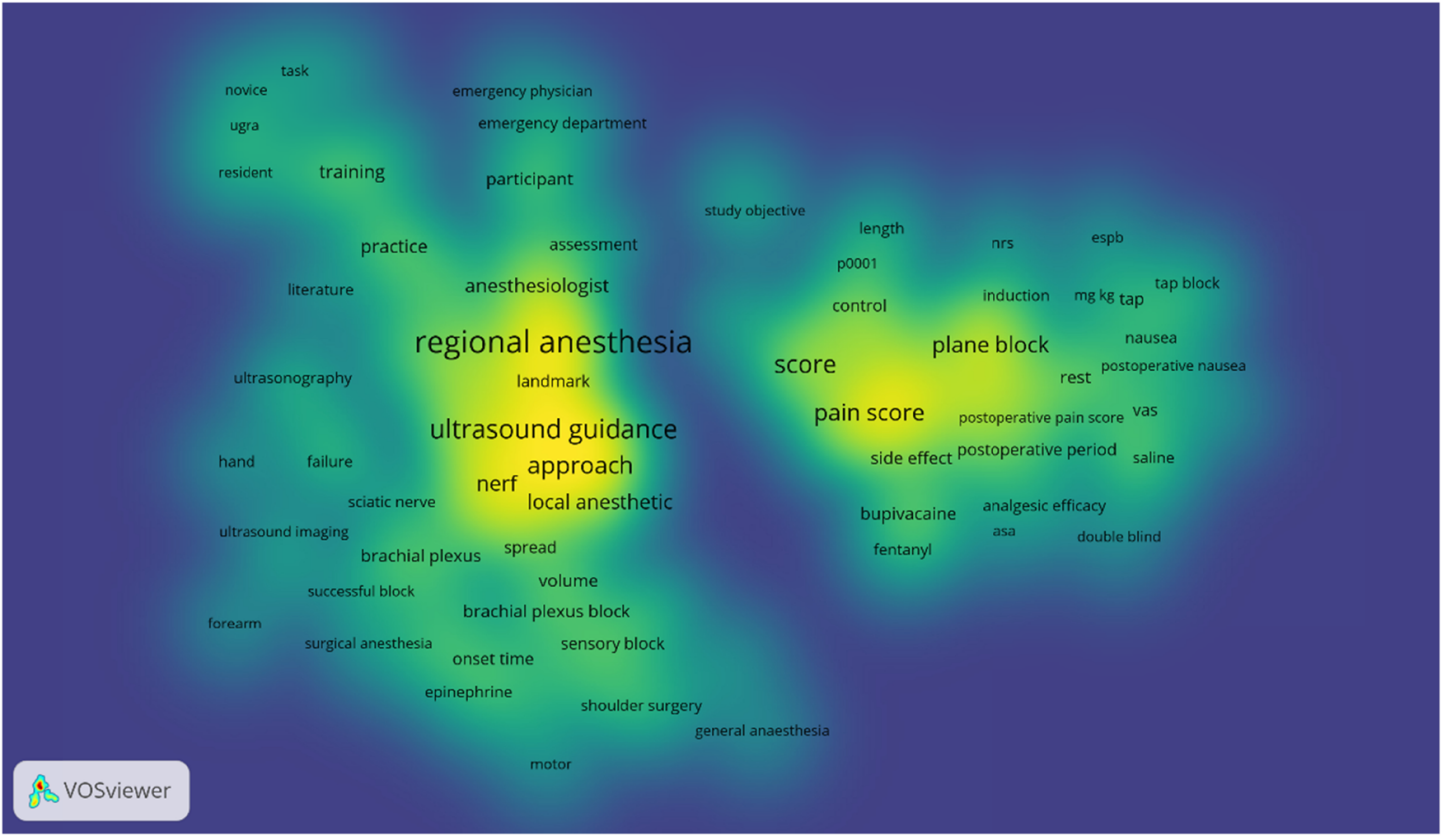
Visualization of hotspot density.

In Figure 5, the VOSviewer software is used to draw the heat cluster graph of keywords and keywords, which shows that the colors of Regional Anesthesia, Ultrasound guidance, Approach, Pain score, and Plane block are yellow, indicating that these keywords have high research hotspots.

### 3.5 Research frontier and evolution path

The concept of research frontier marks the development trajectory and direction of a particular problem area. It includes a range of emerging and dynamic questions, as well as potential areas of research. In this particular area of research, the first published literature appeared in 1994, followed by sporadic and scattered articles in subsequent years. Until the last decade, the number of articles in this area of study increased significantly.

**Figure 6:**
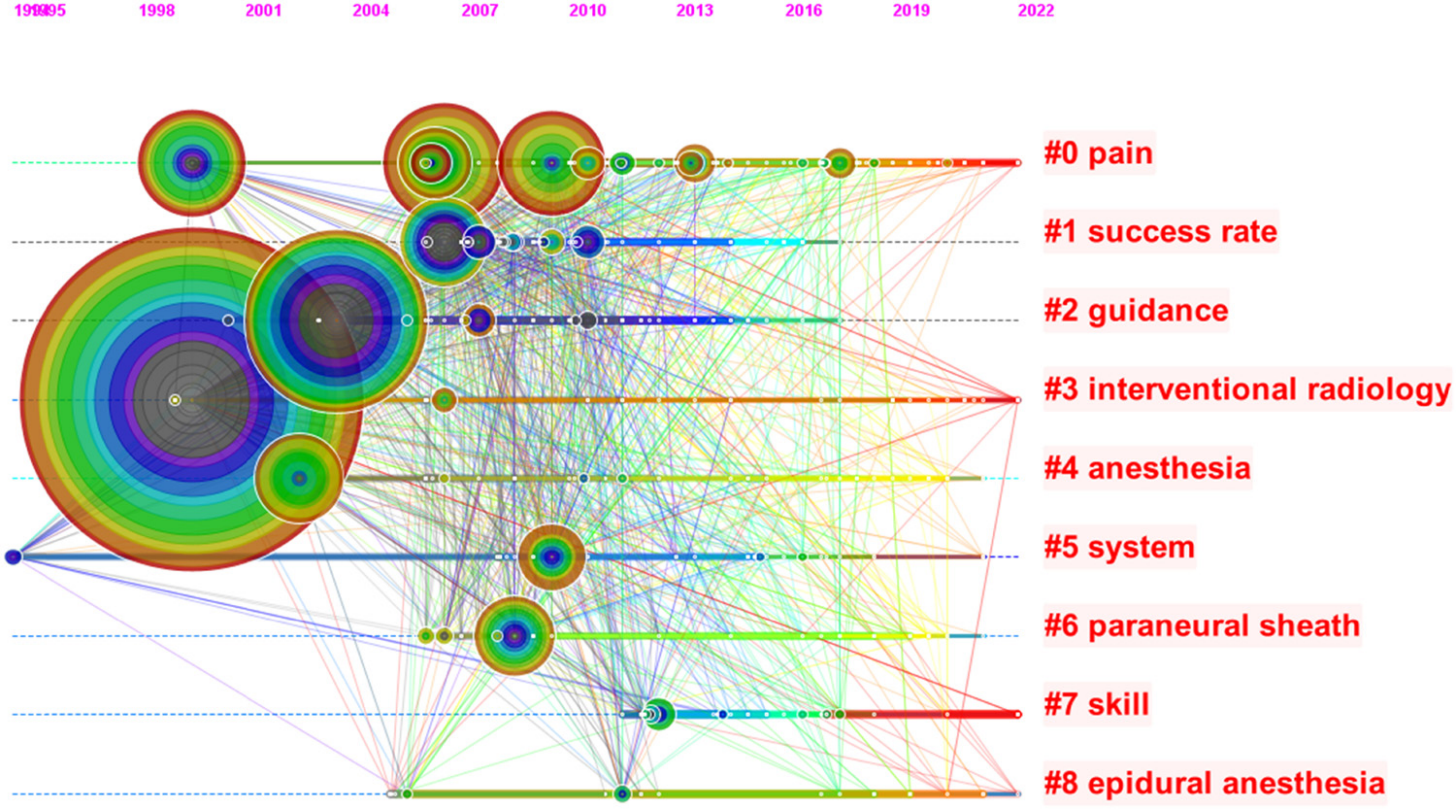
Time chart of keywords.

Figure 6 depicts the temporal evolution of keywords through CiteSpace software, revealing an evolving field of research characterized by evolving topics such as distress, success, and mentoring. It is worth noting that some of the earlier themes, such as regional anesthesia (the most prominent block), have persisted and remain relevant in current research discourse.

### 3.6 Emergent word analysis

**Figure 7:**
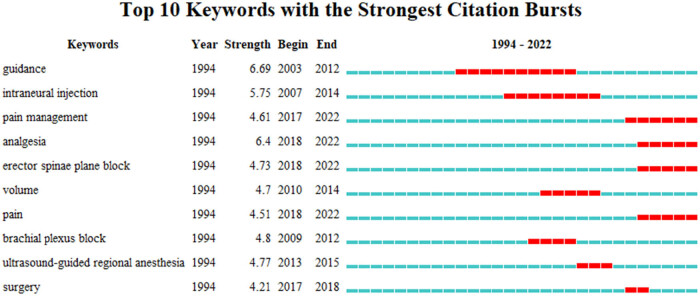
Emergent word diagram.

Emergent words are words with high change rate in a specific period of time. Through the analysis of emergent words, we can understand the new research direction and development trend of current field to a certain extent. Keywords in ultrasound-guided regional anesthesia-related clinical research increase rapidly over time as shown in Figure 7.

### 3.7 Characteristics of national/regional cooperation network

**Figure 8:**
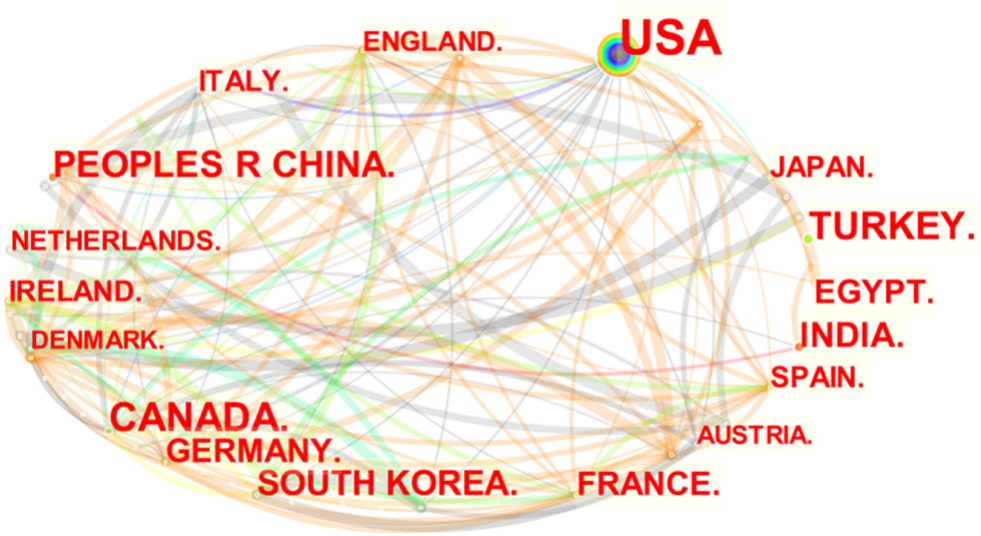
Collaborating countries.

Fifty-one countries/regions participated in the research in this field, and CiteSpace software was used to map the scientific knowledge of national/regional cooperative networks. Figure 8 shows that the nodes in the figure show 17 countries/regions with publication quantity ≥10. The top 10 countries in terms of publications are shown in Table 3. The United States published the most, with 153 articles, followed by Canada, Turkey, China, and South Korea. From the perspective of centrality, the United States, Canada, and Austria occupy the leading position in this research field, and many countries/regions have relatively low academic activity in this field, so it is not shown in the map.

**Table 3:** Top 10 countries and regions in terms of number of publications

	Country	Count	Year		Country	Centrality	Year
1	USA	153	2002	1	USA	0.43	2002
2	Canada	58	2008	2	Canada	0.13	2008
3	Turkey	45	2013	3	Austria	0.11	2006
4	China	38	2015	4	Netherlands	0.07	2008
5	India	32	2010	5	India	0.04	2010
5	South Korea	32	2011	5	Japan	0.04	2009
7	France	27	2010	5	England	0.04	2008
8	Germany	26	2008	5	Belgium	0.04	2010
9	Egypt	25	2014	9	France	0.02	2010
10	Ireland	16	2009	9	Germany	0.02	2008
10	Japan	16	2009				

### 3.8 Characteristics of institutional cooperation network

**Figure 9:**
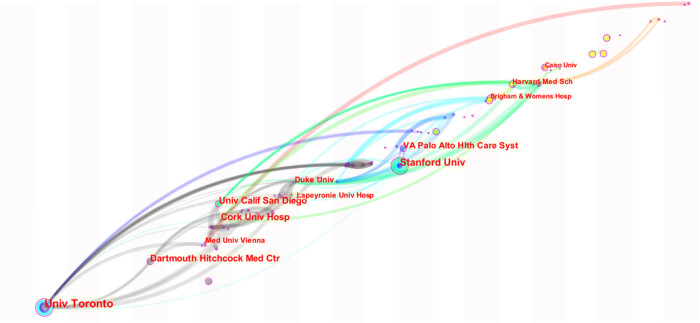
Organization cooperation network.

The collaborative scientific knowledge map of institutions was drawn with CiteSpace software (Figure 9). A total of 377 institutions participated in the research in this field, and the top 10 institutions with publications are shown in Table 4. The nodes in the figure are 12 institutions with publications ≥5.

**Table 4:** Top 10 institutions in terms of number of publications

	Institution	Count	Year
1	Univ Toronto	20	2003
2	Stanford Univ	18	2012
3	Cork Univ Hosp	12	2009
4	Dartmouth Hitchcock Med Ctr	10	2006
4	Univ Calif San Diego	10	2008
6	VA Palo Alto Hlth Care Syst	9	2013
7	Duke Univ	7	2010
8	Lapeyronie Univ Hosp	6	2010
8	Med Univ Vienna	6	2007
8	Harvard Med Sch	6	2017

Node size and data confirm that Stanford University, the University of Toronto and Cork University Hospital have published articles in the top three. The map of institutional collaborative scientific knowledge shows the highest mediating centrality among University Toronto, Cork University Hospital and University California San Diego. The centrality of other institutions is mostly 0, indicating the low degree of cooperation among institutions in this field.

### 3.9 Co-author analysis

**Figure 10:**
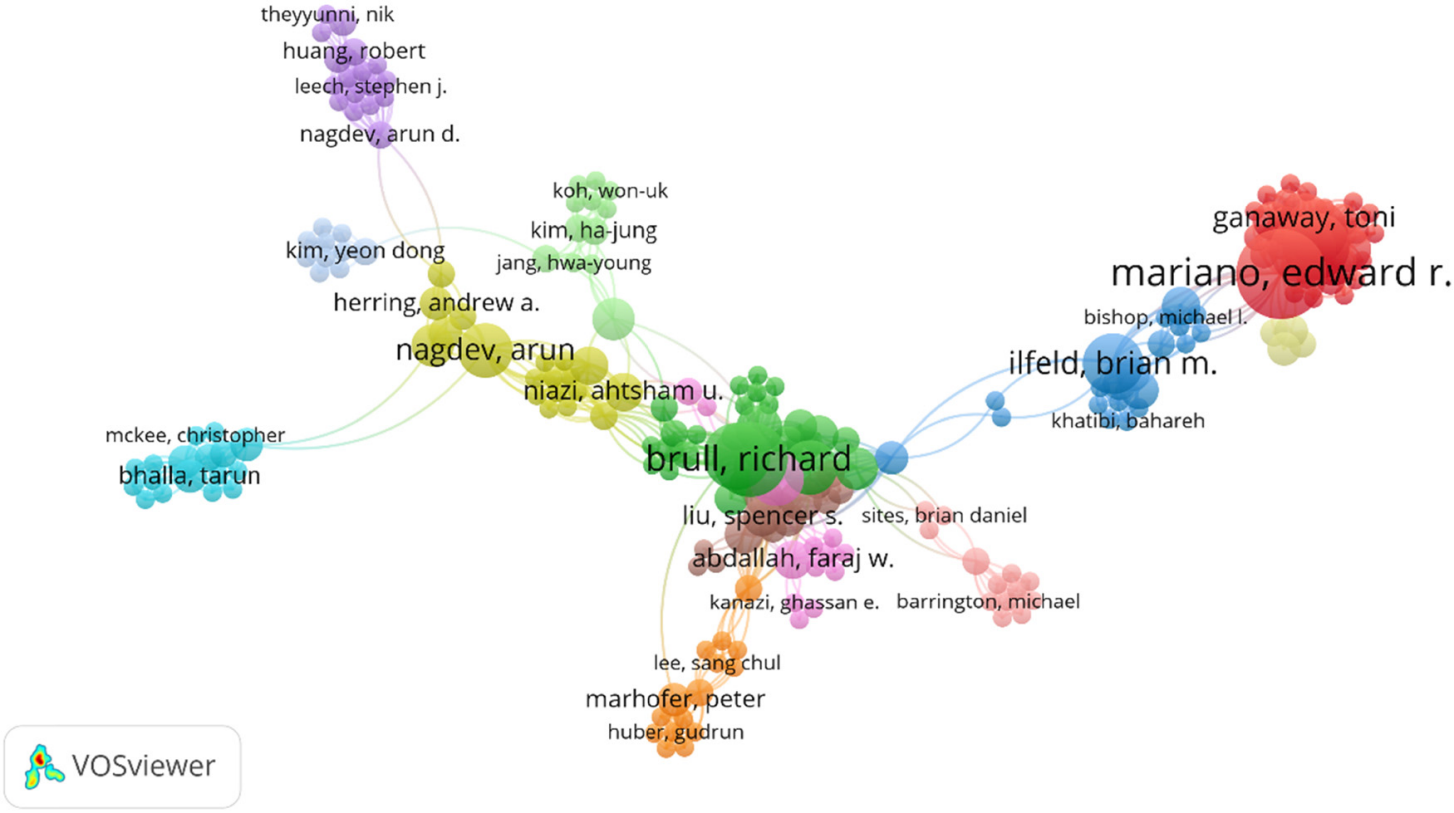
Author cluster map.

VOSviewer was used to map the collaborative scientific knowledge of authors. The 212 authors included in the literature were clustered and 13 obvious author clusters were mainly formed. As shown in Figure 10, there are certain cooperative relationships among all clusters, but most of the cooperative relationships are mainly within clusters.

The top 10 authors in terms of publications are shown in Table 5. The author who published the most articles in the field of regional anesthesia under ultrasound guidance was CHAN V, who published 24 related literatures. The most cited author is MARHOFER P, whose studies and publications in the field of ultrasound-guided regional anesthesia have been cited 150 times.

**Table 5:** Top 10 author in terms of number of publications

	Author	Count	Year		Cited author	Count	Year
1	Chan V	24	2003	1	Marhofer P	150	2005
2	Mariano E	18	2009	2	Sites B	148	2006
3	Brull R	14	2007	3	Neal J	99	2008
4	Kim T	10	2014	4	Chin K	80	2010
5	Howard S	9	2014	5	Chan V	74	2006
6	Kim J	8	2015	6	Perlas A	67	2007
6	Harrison T	8	2014	7	Fredrickson M	64	2009
7	Sites B	7	2006	8	Liu S	55	2010
7	Abbas S	7	2007	8	Gray A	55	2005
7	Marhofer P	7	2006	10	Casati A	53	2006

### 3.10 Co-citation characteristics of journals

**Figure 11:**
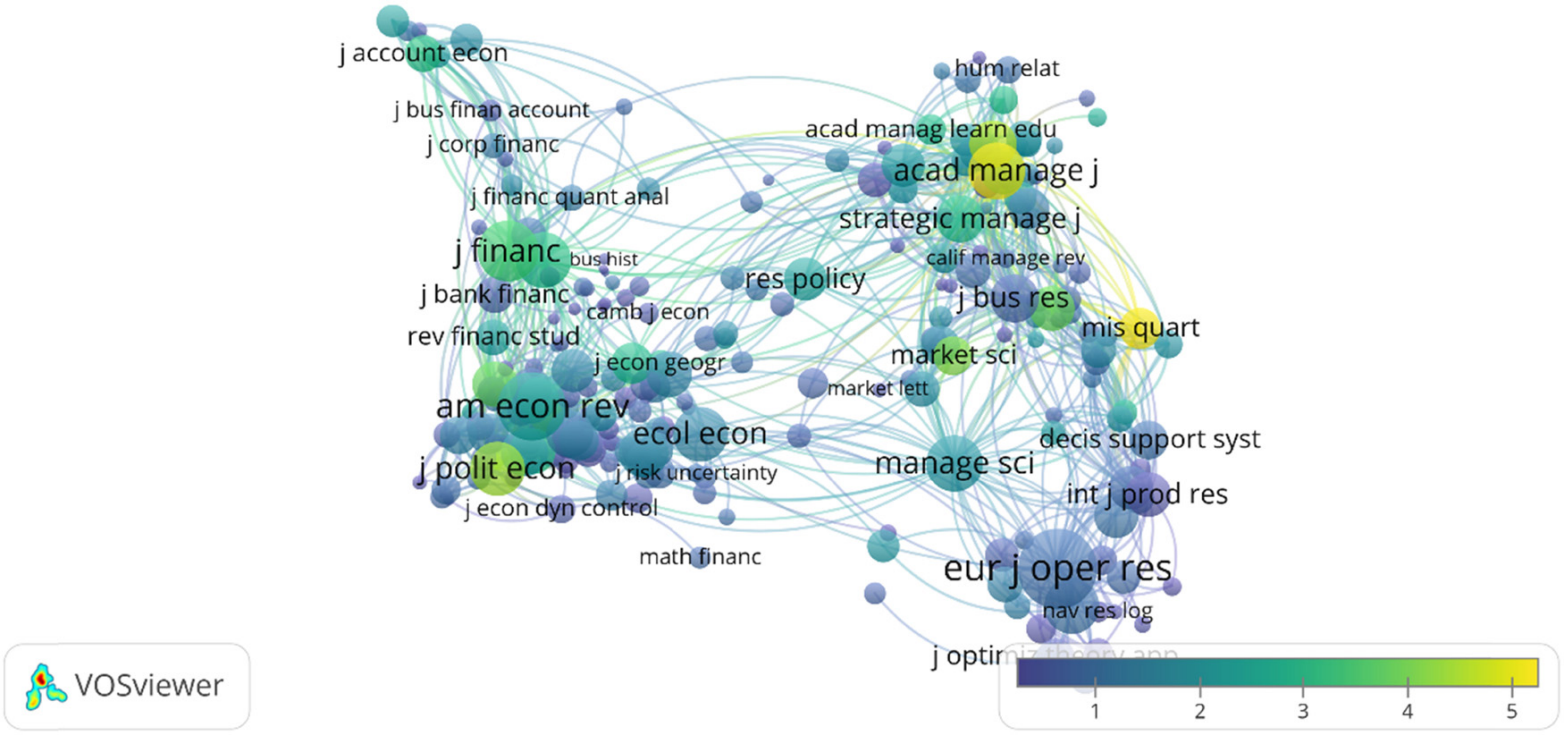
Co-citation characteristics of journals.

The VOSviewer software was used to create a journal overlay visualization map, and 232 journals reported research in this area. As shown in Figure 11, the closer the node color is to yellow, the higher the impact factor of the journal is.

Table 6 shows the top 10 journals with the most published and cited articles in the field of Regional Anesthesia under ultrasound. Regional Anesthesia and Pain Medicine has published 77 articles and has been cited 472 times. The second and third most cited are Anesthesia and Analgesia and the British Journal of Anaesthesia, with 418 and 378 citations respectively. The second and third places were Veterinary Anaesthesia and Analgesia and Anesthesia and Analgesia, with 26 and 24 papers published respectively.

**Table 6:** Top 10 journal in terms of number of publications

	Cited journal	Count	HI	IF		Journal	Count	HI	IF
1	*Regional Anesthesia and Pain Medicine*	476	109	3.129	1	*Regional Anesthesia and Pain Medicine*	71	109	3.129
2	*Anesthesia and Analgesia*	418	201	2.659	2	*Pediatric Anesthesia*	24	82	2.282
3	*British Journal of Anaesthesia*	378	181	4.328	3	*Journal of Clinical Anesthesia*	22	69	1.998
4	*Anesthesiology*	324	234	2.64	4	*Anesthesia and Analgesia*	21	201	2.659
5	*Anaesthesia*	275	117	2.775	5	*Anaesthesia*	20	117	2.775
6	*Acta Anaesthesiologica Scandinavica*	176	107	1.542	5	*Journal of Ultrasound in Medicine*	20	91	1.922
7	*Journal of Clinical Anesthesia*	139	69	1.998	7	*Journal of Anesthesia*	18	44	1.473
8	*Canadian Journal of Anesthesia*	119	97	3.199	8	*Acta Anaesthesiologica Scandinavica*	14	107	1.542
9	*European Journal of Anaesthesiology*	114	76	2.17	9	*British Journal of Anaesthesia*	12	181	4.328
9	*Journal of Anesthesia*	114	44	1.473	9	*Medicine*	12	148	1.644

## 4 Discussion

Bibliometric analysis is an emerging tool developed in recent years to rapidly explore research structures and trends in targeted topics through statistical methods and visualizations [11]. By identifying relevant nodes, useful information can be extracted from a large amount of information through software analysis [12]. Commonly used bibliometric software, including CiteSpace [13], VOSviewer [14], bibExcel [15], and HistCite [16], are all used for scientific bibliometric analysis. CiteSpace and VOSviewer are the most popular. CiteSpace software can intuitively display the location and size of each node in the knowledge network. By selecting different functions, it can analyze the source regions, researchers, research hotspots, and their evolution of research literatures in related fields [17]. VOSviewer is a software that takes a holistic view of all research subjects and explores research topics across an entire field by selecting different perspectives [10]. In this study, VOSviewer and CiteSpace are used together to give full play to their different advantages.

Anesthesia is one of the most important perioperative operations of surgical patients, and regional anesthesia is an important part of it. Regional anesthesia features the highest analgesic technique [18]. Its key benefits are better analgesia, reduced stress responses, fewer systemic opioid side effects, protection against chronic pain, and improved quality of life [19]. Ultrasound-guided assistance helps to assist anesthesiologists in using regional anesthesia as a safe and effective alternative to traditional general anesthesia [20].

In this study, literature metrology was employed for the visual analysis and examination of 570 previously published non-review articles pertaining to ultrasonics-guided regional anesthesia. Research in this field has been conducted over the course of the 20th and 21st centuries, with an average of 18 citations per included paper. Notably, after 2007, there was an explosive growth in the number of studies, culminating in a peak in 2021. Keyword analysis showed that “regional anesthesia”, “guidance”, and “pain” appeared the most frequently and were most closely related to other words. Regional anesthesia, ultrasound guidance, approach, pain score, and plane block are the five keywords with high research hotspots. The hot research topics are Erector Spinae plane block, Serratus Plane block, Brachial Plexus block, postoperative pain, and Axillary block; clinical research can make its own efforts in these aspects.

The United States has published the most articles in the field of regional anesthesia under the guidance of ultrasound. It is also the country at the center of this research field and has the closest research cooperation and exchange with other countries and regions. There is less cooperation among institutions, and Stanford University, University of Toronto, and Cork Univ Hospital published the most literature. Regional Anesthesia and Pain Medicine are the most widely published and cited journal in the field of ultrasonics-guided Regional Anesthesia. Experts and scholars interested in the subject can find more new developments in this journal.

Visual analysis conducted using CiteSpace and VOSviewer software, while valuable, cannot entirely replace systematic retrieval, and it does have certain limitations that need to be addressed. First, the literature collected for this study spans from 1994 to 2022. Given the continuous updating of literature databases, there may be some discrepancy between the retrieval results of this study and the actual number of relevant publications. Second, this study exclusively includes articles, which constitute a single type of literature. Additionally, the quality of the collected literature may vary significantly, potentially impacting the credibility of the visual analysis. Nevertheless, it is important to recognize that visual analysis based on the available literature serves as a foundational resource for scholars seeking to gain rapid insights into the realm of ultrasonics-guided regional anesthesia.

The exploration of ultrasound-guided regional anesthesia holds significant research value and promises extensive practical applications. Research endeavors within this field exhibit a consistent year-on-year increase. By leveraging CiteSpace and VOSviewer software, we have visualized and analyzed the trends and distinctive features of ultrasound-guided regional anesthesia research. This visual analysis offers valuable bibliometric insights, providing researchers with a solid foundation to delve deeper into this field and advance their understanding.
